# Quantifying responses of climatic and anthropogenic factors on vegetation normalized difference vegetation index variabilities over Yellow River Basin, China

**DOI:** 10.3389/fpls.2026.1756685

**Published:** 2026-02-27

**Authors:** Zhilei Yu, Ligen Wang, Junfei Yan, Zhe Yuan, Xiangyang Zhang

**Affiliations:** 1 School of Water Conservancy and Transportation, Zhengzhou University, Zhengzhou, China; 2 Key Laboratory of Water Management and Water Security for Yellow River Basin, Ministry of Water Resources (under construction), Zhengzhou, China; 3 Songxian Institute of Soil and Water Conservation, Songxian Water Resources Bureau, Luoyang, Henan, China; 4 Changjiang River Scientific Research Institute, Changjiang Water Resources Commission of the Ministry of Water Resources of China, Wuhan, China

**Keywords:** anthropogenic factors, climatic factors, remote sensing, sustainable environmental development, vegetation NDVI

## Abstract

**Introduction:**

Climatic change and anthropogenic activities have substantially influenced vegetation distribution in recent decades. However, identifying the dominant driving factors of vegetation variation remains challenging.

**Methods:**

This study investigated vegetation dynamics and quantified their responses to climatic factors (effective precipitation (EFPR) and active accumulated temperature ≥10 °C (ACTE)) and anthropogenic activities (urbanization, afforestation, and cultivation) for the Yellow River Basin (YRB).

**Results:**

The results showed that the YRB experienced vegetation greening in NDVI (as measured by normalized difference vegetation index, NDVI) during the period from 2001 to 2020. More than 90% of the regions in the YRB showed an increasing NDVI trend, with mean rates of 0.055 per decade. In regions with significant (*p* < 0.05) variations in NDVI, ERPE, ACTE, and anthropogenic activities contributed to vegetation dynamics at rates of 0.012 per decade, 0.007 per decade, and 0.036 per decade, respectively. Contributions of climatic and anthropogenic factors accounted for 35% and 65% of the total NDVI variations, respectively.

**Discussion:**

Both climatic and anthropogenic factors drove the vegetation growth. In the alpine source regions of the Yellow river, climatic factors were the primary drivers of significant NDVI change. In the MB_YRB (middle basin) and the LB_YRB (lower basin), human activities were the main factors driving vegetation greening. Only in areas with urban agglomeration, such as Xi’an, Zhengzhou and Xi’ning cities, were anthropogenic activities associated with vegetation browning.

## 1 Introduction

Vegetation is of importance to connect ecosystems and climatic changes and anthropogenic activities (Li and Wang, 2025). Climate changes and human activities affect the growth, function, and distribution pattern of vegetation in the terrestrial ecosystem (Li et al., 2022; Su et al., 2025). Understanding the spatio-temporal dynamics of vegetation is essential to provide a theoretical basis for ecological protection and sustainable development (Li et al., 2021; Zhang et al., 2020; Zhao et al., 2020). However, the mechanisms driving these dynamics exhibit profound spatial heterogeneity across ecologically fragile regions, necessitating a more granular approach to disentangle the complex interplay between specific climatic constraints and human interventions (Liu et al., 2022; Xu et al., 2024). Numerous studies have explored vegetation dynamics and the coupled relationships between vegetation and climatic factors, especially precipitation and temperature. Since 2000, global or regional vegetation has greened significantly (Bao et al., 2025; Wang et al., 2022b), especially in bare and sparsely vegetated regions (Bao et al., 2025). Precipitation and temperature have been identified as the key factors driving vegetation growth directly and significantly (Wang et al., 2021). However, while the normalized difference vegetation index (NDVI) is widely used to characterize these trends, traditional climatic drivers often fail to fully explain its spatial–temporal variations (Cui et al., 2022). A critical research gap remains in identifying the direct physiological constraints on NDVI; specifically, it is not gross temperature but the active accumulated temperature (ACTE) that governs plant thermal niches and phenological cycles (Gu et al., 2022; Li and Zhong, 2024), and it is not total precipitation but effective precipitation (EFPR) that determines the actual water available for plant metabolic activities (Wang et al., 2022a; Wang and Collins, 2024). Furthermore, the complex interplay between these water–heat factors often masks their individual effects. Simple correlation analyses are insufficient to isolate these drivers, necessitating more rigorous approaches (Liu et al., 2018a) such as partial and multiple correlation methods to quantify the independent influence of ACTE and EFPR on NDVI (Yan et al., 2025). In addition, land cover changes (such as urbanization and afforestation of anthropogenic activities) have altered the dynamics of terrestrial vegetation (Hao and Sun, 2021; Jin et al., 2022). Most existing studies have focused on the sensitivity of vegetation activities to driving factors, whereas quantitative assessments of vegetation responses to both climatic and anthropogenic factors remain limited. Consequently, it remains difficult to provide a solid theoretical basis to understand interactions between terrestrial ecosystems and environmental factors.

Therefore, it was crucial to understand spatio-temporal variations of vegetation patterns and their relationship with climatic changes and anthropogenic activities (Shrestha et al., 2024; Xue et al., 2021). As an ecological barrier in northern China, the vegetation of the Yellow River Basin (YRB) was extremely sensitive to environmental changes (Feng et al., 2021). Accordingly, we investigated the effects of water–heat conditions and human activities on terrestrial vegetation variations over YRB. Differently from the previous studies, we selected ACTE and EFPR as climatic factors and nighttime light, forestlands, and croplands as anthropogenic factors to (a) identify the spatio-temporal patterns of terrestrial vegetation in the YRB over the past 20 years (2001–2020), (b) quantify the contributions of climatic and anthropogenic factors to terrestrial vegetation activities in the YRB, respectively, and (c) explore the main driving forces affecting the variations and spatial patterns of vegetation.

## 2 Overview of the study area

**Figure 1:**
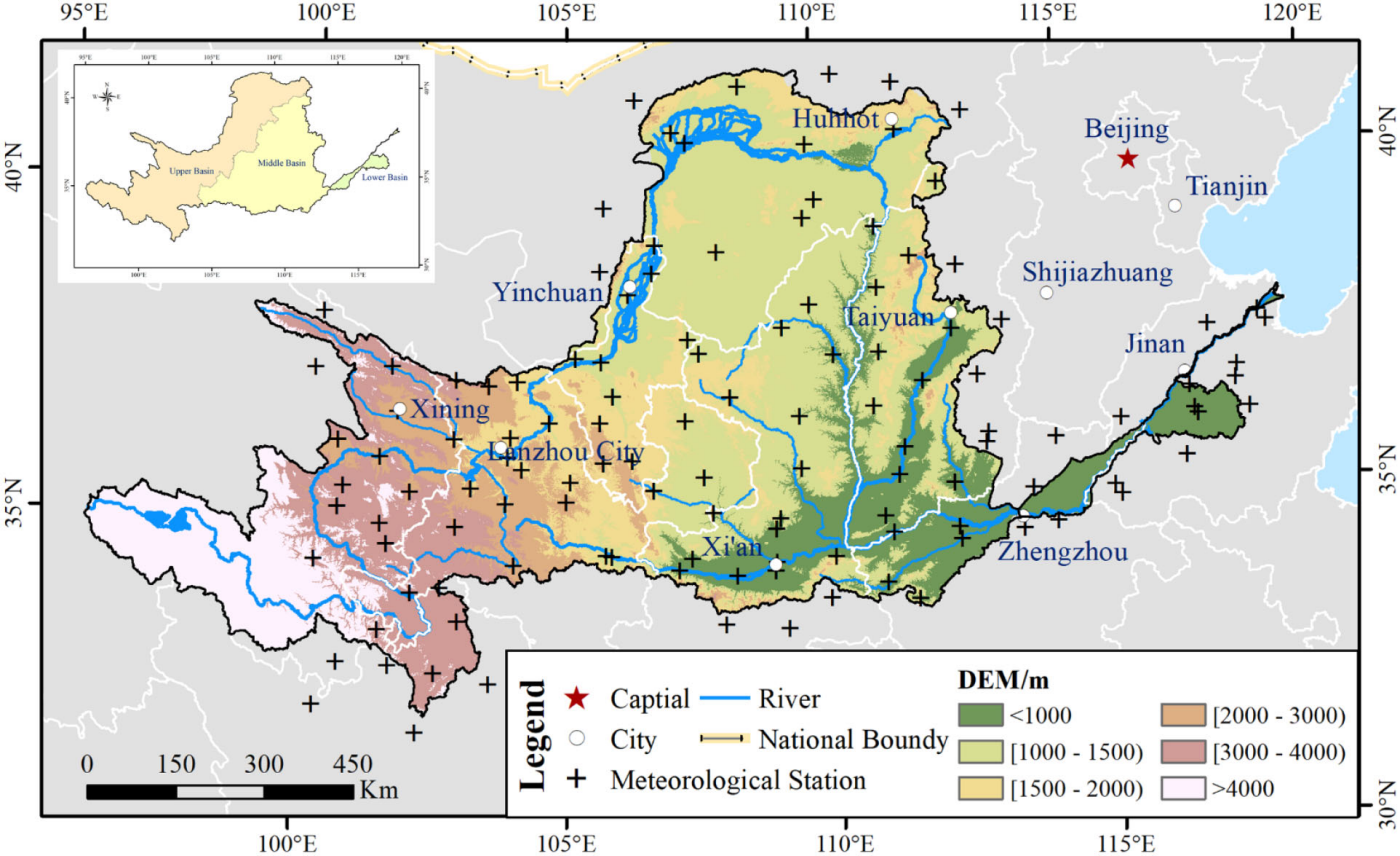
Location of the Yellow River Basin (YRB) and the Upper Basin (UB_YRB), Middle Basin (MB_YRB), and Lower Basin (LB_YRB) of YRB.

**Figure 2:**
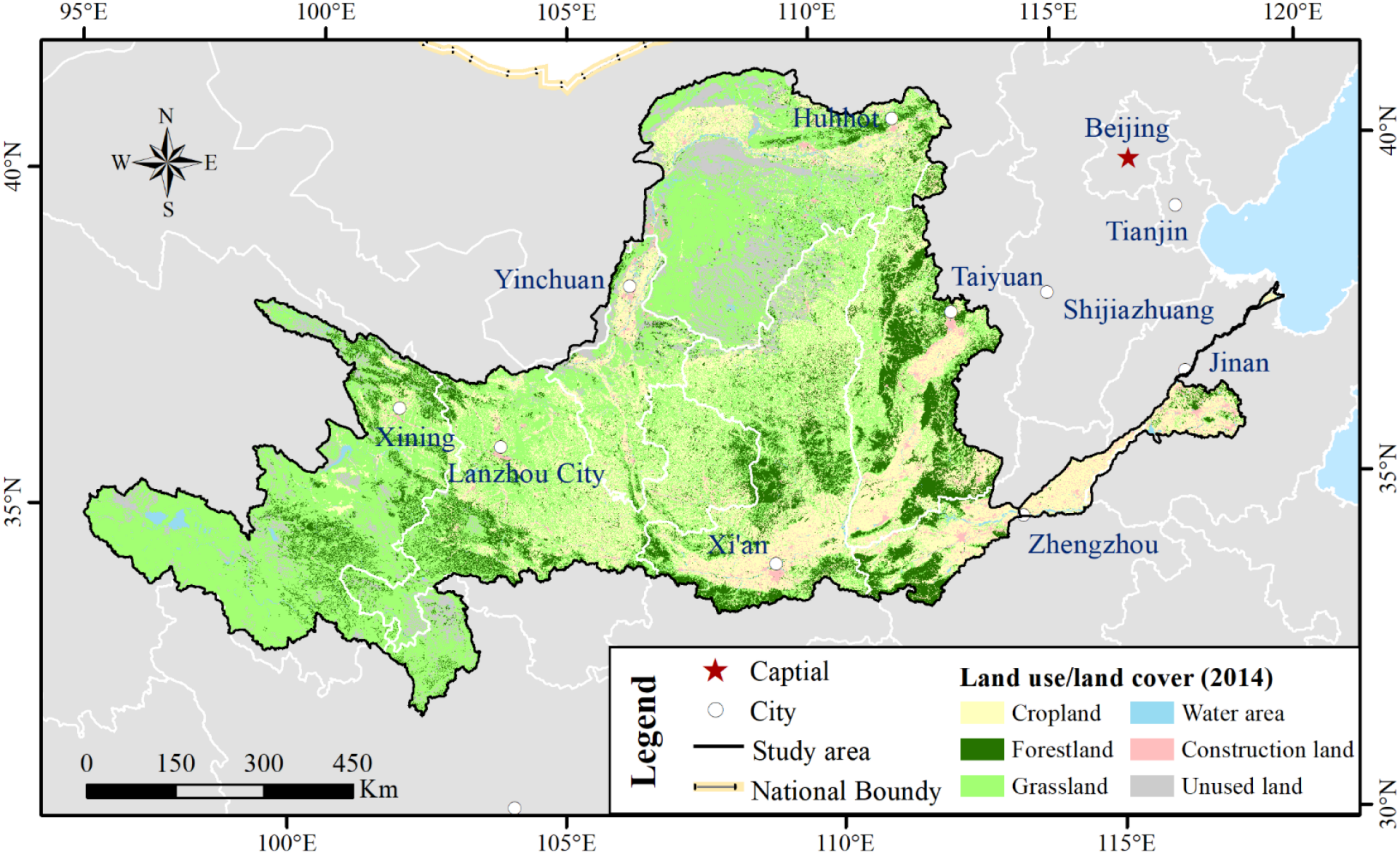
Spatial distribution of the primary land use/land cover types over YRB.

The YRB is located at 32°9′32″ N–41°50′19″ N, 95°53′48″ E–119°3′56″ E with a total basin area of 7.9 × 10^5^ km^2^ (**Figure 1**). The YRB is the most extremely important basin in the middle of eastern China. The Yellow River originates from the Qinghai–Tibet Plateau, with the YRB covering nine provinces. The terrain of the YRB is higher in the west and is relatively flat in the east. The altitude of the entire basin ranges from 1 to 6,188 m. It spans across the Qinghai–Tibet Plateau, the Inner Mongolia Plateau, the Loess Plateau, and the Huang-Huai-Hai Plain from west to east (Yang et al., 2021). Most areas of the YRB are characterized by the temperate continental monsoon climate, which belongs to the typical East Asian monsoon climate zone (He et al., 2021; Zhang et al., 2020). The YRB has arid, semi-arid, and semi-humid climates from the northwest to southeast (Gu et al., 2021a). The main vegetation cover categories include crops, forests, grasses, water area, construction land, and other unused land in the YRB (**Figure 2**). **Figure 2** shows the spatial characteristics of primary vegetation cover, which is obtained from the Resource and Environment Science and Data Center, Institute of Geographic Sciences and Natural Resources Research, CAS (http://www.resdc.cn). The watershed data were obtained from the National Earth System Science Data Center, National Science and Technology Infrastructure of China (http://www.geodata.cn).

## 3 Materials and methods

### 3.1 Datasets

#### 3.1.1 NDVI data

The normalized difference vegetation index (NDVI) is widely used to represent vegetation coverage level. It was derived from Terra MODIS vegetation indices MOS13Q1.006 (https://appeears.earthdatacloud.nasa.gov/), with a spatial resolution of 250 × 250 m from 2001 to 2020. We used the maximum value composite (MVC) method and the ArcGIS 10.2 platform to derive the annual NDVI for the YRB.

#### 3.1.2 ACTE and EFPR data

We used the data on daily precipitation and temperature of the 100 (93) meteorological stations during the period from 2001 to 2020 over YRB, which were provided by the National Meteorological Information Center (http://data.cma.cn/). The inverse distance weighted (IDW) method and AML language were applied to calculate the annual precipitation and temperature. Subsequently, we calculated the ACTE and EFPR (Yan et al., 2013, 2011) to characterize the effective hydro-thermal conditions—specifically:

(a) The ACTE was calculated as the accumulated daily mean temperature ≥10 °C. This threshold was selected because 10 °C is widely recognized as the biological zero point for active photosynthesis in temperate vegetation, representing the thermal energy actually available for growth better than the mean temperature (PIAO et al., 2006).

(b) Effective precipitation (EFPR) refers to the portion of rainfall that infiltrates the soil and is available for crop use, primarily stored in the root zone. In this study, we used the method proposed by Yan et al. to calculate EFPR (Nie et al., 2019; Yan et al., 2013).


$$EFPR=\sum_{i=1}^{n}\mu P_{i}$$

where *P_i_* is the daily precipitation for day *i* and *μ* is the effective utilization coefficient of precipitation. The different monthly precipitation corresponds to different *μ* values, as shown in **Table 1**.

**Table 1:** Effective utilization coefficient of precipitation.

Monthly precipitation/mm	<5	5–30	30–50	50–100	100–150	>150
μ	1.00	0.85	0.80	0.70	0.58	0.48

#### 3.1.3 Nighttime light data

In the present study, we used DMSP-OLS (Defense Meteorological Satellite Program Operational Linescan System) dataset (http://www.resdc.cn/) to investigate the spatial changes in human activities across the YRB from the 2000 to 2013. The digital number (DN) value ranged from 0 to 63 that indicated the light variations by cities and human activities. Although the time series of DMSP-OLS data covered only 14 years, the nighttime light data remain meaningful to explain the spatial variations of rapid urbanization (Yuan et al., 2021). ArcGIS 10.2 software was applied to extract and project the data of the YRB to mate the NDVI data.

#### 3.1.4 Other data

We integrated the annual statistical data in agriculture and forestry by the National Bureau of Statistics of China (https://data.stats.gov.cn/), land use data (2001 to 2020) with a spatial resolution of 1 × 1 km from RESDC (http://www.resdc.cn/), and anthropogenic heat flux (AHF) of land surface (spatial resolution 500 m × 500 m) (http://data.tpdc.ac.cn/zh-hans/) to analyze the causes of NDVI variation. The agriculture and forestry statistical data included the harvested area, effective irrigation area, total food production, fertilizer use amount, forest area, forest cover rate, and accumulated afforested area from 2004 to 2020. Land use types were divided into six categories, including cropland (cultivated land), forestland, grassland, watershed (water area), construction land, and barren land (unused land).

### 3.2 Methods

#### 3.2.1 Linear regression and the trend

We used the linear regression method to eliminate the trend rate of essential factors (Hu and Daorina and Hu, 2019), including the NDVI, ACTE, and EFPR, the formula of which is as follows:


$$Slope=\frac{n\times \sum_{i=1}^{n}i\times X_{i}-(\sum_{i=1}^{n}i)\times (\sum_{i=1}^{n}X_{i})}{n\times \sum_{i=1}^{n}i^{2}-{\left(\sum_{i=1}^{n}i\right)}^{2}}$$

where *slope* represents the estimated linear trend of *X_i_* change during the period of 2001 to 2020. *X_i_* means the value of NDVI, ACTE, or EFPR for the *i*-th year (*i* = 1, 2, …, *n*). The *slope >*0 illustrates the variables’ increasing trends, while *slope <*0 denotes decreasing trends.

We used *F*-test to detect the significance of the variation trend, and the *F*-value equation is as follows:


$$F=\frac{(n-2)\times \sum_{i=1}^{n}{\left(\hat{X_{i}}-\bar{X}\right)}^{2}}{\sum_{i=1}^{n}{\left(X_{i}-\hat{X_{i}}\right)}^{2}}$$

where $\hat{X_{i}}$ represents the variable regression value of the *i*^th^ year, and $\bar{X}$ shows the average value of variables from 2001 to 2020.

#### 3.2.2 Analysis of the partial correlation and multiple correlation

To estimate the response of the NDVI to the ACTE and EFPR in the YRB, we use the partial correlation and multiple correlation methods to calculate the correlation between NDVI and climatic forces (Yuan et al., 2021). The partial correlation coefficient is used to quantify relationships between NDVI and EFPR or ACTE. The multiple correlation coefficient is utilized to estimate the relationship between NDVI and climatic factor. The correlation coefficient equations are as follows:

Partial correlation coefficient.


$$r_{zx,y}=\frac{r_{zx}-r_{zy}-r_{xy}}{\sqrt{\left(1-r_{zy})(1-r_{xy}\right)}}$$

where *r_zx,y_* denotes the partial correlation coefficient of variables *x* and *z*, while variable *y* is constant. *r_zx_*, *r_zy_*, and *r_xy_* represent correlation coefficients of the variables *x* and *z*, *y* and *z*, and *x* and *y*, respectively. *t*-test is used to estimate the *r_zx,y_* significance as follows:


$$t=\frac{r_{zx,y}}{\sqrt{\left(1-r_{zx,y}^{2}\right)}}\sqrt{n-m-1}$$

where *n* and *m* are the samples’ and independent variables’ number, respectively.

multiple correlation coefficient.


$$R_{z,xy}=\sqrt{1-(1-r_{zx}^{2})(1-r_{zy,x}^{2})}$$

where *R_z,xy_* describes the multiple correlation coefficient of variable *z*, while variables *x* and *y* are independent. *F*-test is utilized to evaluate the *R_z,xy_* significance as follows:


$$F=\frac{R_{z,xy}^{2}}{1-R_{z,xy}^{2}}\times \frac{n-k-1}{k}$$

where *n* and *k* are the samples’ and independent variables’ number, respectively.

#### 3.2.3 Definition climatic driving factors on NDVI changes

**Table 2** demonstrates the classification criteria, which were used to estimate the relative contribution of different elements on the NDVI variations (Yuan et al., 2021). F_CLI_ indicates the multiple correlation between major climatic factors and NDVI; C_EPR_ or C_ACT_ shows the partial correlation between EFPR or ACTE and NDVI.

**Table 2:** Classification criteria for dominant climate factors’ definition.

Driving factors		Criteria
Effective precipitation (EFPR)		F_CLI_ < F_0.1_, C_EPR_ < C_0.05_, C_ACT_ ≥ C_0.05_
Accumulated temperature (ACTE)		F_CLI_ < F_0.1_, C_EPR_ ≥ C_0.05_, C_ACT_ < C_0.05_
Major climatic factors	Weak	F_CLI_ < F_0.1_, C_EPR_ ≥ C_0.05_, C_ACT_ > C_0.05_
	Strong	F_CLI_ < F_0.1_, C_EPR_ < C_0.05_, C_ACT_ < C_0.05_

#### 3.2.4 Contribution of the climate changes and human activities on NDVI

The positive NDVI slope indicates that NDVI increased and vice versa. Climatic and anthropogenic factors affected the NDVI changes. Thus, we used $\epsilon_{1}\frac{dEFPR}{dt}$, $\epsilon_{2}\frac{dACTE}{dt}$, and RES to quantify the relative impacts of climatic and anthropogenic factors on NDVI variations in the YRB. The equations are as follows:


$$\frac{dNDVI}{dt}=\epsilon_{1}\frac{dEFPR}{dt}+\epsilon_{2}\frac{dACTE}{dt}+RES$$

where $\epsilon_{1}\frac{dEFPR}{dt}$, $\epsilon_{2}\frac{dACTE}{dt}$, and RES present the dynamic contribution of the EFPR, ACTE, and human activities affecting NDVI variation. $\frac{dEFPR}{dt}$ and $\frac{dACTE}{dt}$ show the time rate of the EFPR and ACTE changes covering the period 2001 to 2020, which were obtained from Section 3.2.1.

Increasing caused by climatic and anthropogenic factors, GCH.

Increasing caused by climatic factors, GC.

Increasing caused by anthropogenic factors, GH.

Decreasing caused by climatic and anthropogenic factors, BCH.

Decreasing caused by climatic factors, BC.

Decreasing caused by anthropogenic factors, BH.

In **Table 3**, we calculate the contributions of climate changes and human activities on NDVI. *NDVI_SLOPE_* indicates the linear trend of NDVI from 2001 to 2020. C*NDVI_SLOPE_* presents the dynamic contribution of EFPR and ACTE. H*NDVI_SLOPE_* means anthropogenic contribution on the NDVI changes.

**Table 3:** Contributions of the climate changes and human activities on NDVI.

Scenarios	NDVI slope	CNDVI	HNDVI	Contribution	
				Climate (%)	Human (%)
GCH	> 0	> 0	> 0	$\frac{CNDVI_{SLOPE}}{NDVI_{SLOPE}}$	$\frac{HNDVI_{SLOPE}}{NDVI_{SLOPE}}$
GC	> 0	> 0	< 0	100	0
GH	> 0	< 0	> 0	0	100
BCH	< 0	< 0	< 0	$\frac{CNDVI_{SLOPE}}{NDVI_{SLOPE}}$	$\frac{HNDVI_{SLOPE}}{NDVI_{SLOPE}}$
BC	< 0	< 0	> 0	100	0
BH	< 0	> 0	< 0	0	100


$$CNDVI_{SLOPE}=\epsilon_{1}\frac{dEFPR}{dt}+\epsilon_{2}\frac{dACTE}{dt}$$

$$HNDVI_{SLOPE}=RES$$

## 4 Results

### 4.1 Spatial–temporal variation of annual NDVI

#### 4.1.1 Inter-annual variations of NDVI

**Figure 3:**
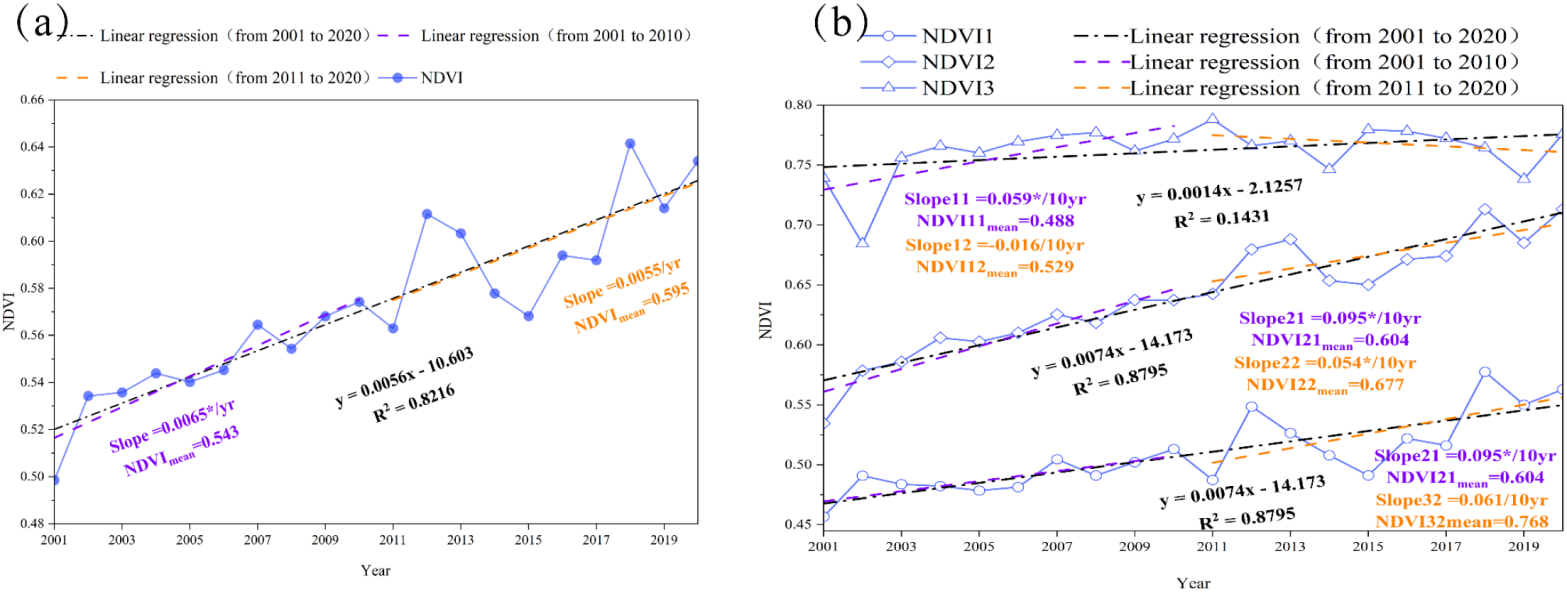
Inter-annual variations and trend magnitudes of NDVI in the Yellow River Basin (YRB) and its sub-basins during different periods. **(a)** Temporal changes in NDVI and corresponding trend slopes for the entire basin from 2001 to 2020. **(b)** Multi-year mean NDVI and NDVI trends for the upper basin (UB_YRB), middle basin (MB_YRB), and lower basin (LB_YRB). The numbers 1, 2, and 3 represent the changes over the UB_YRB (upper basin), MB_YRB (middle basin), and LB_YRB (lower basin), respectively. The numbers 11 and 12 display the variations in stages 1 and 2 over UB_YRB. The numbers 21 and 22 reveal the characteristics during 2001 to 2010 and from 2011 to 2020 in the MB_YRB. The numbers 31 and 32 show the changes from 2001 to 2010 and from 2011 to 2020 over LB_YRB.

**Figure 3a** shows the inter-annual variability of NDVI over the YRB, presenting an increasing trend for the period from 2001 to 2020 (*slope* = 0.056 per decade, *p* < 0.05). The tendency magnitudes of NDVI variations were 0.065 per decade (*p* < 0.05) during the period from 2001 to 2010 and 0.055 per decade during the period from 2011 to 2020, respectively, indicating a relatively strong positive trend before 2010. However, the mean NDVI was 0.600 from 2011 to 2020, increasing by approximately 10% compared to the multiyear average NDVI (0.546) for the period from 2001 to 2010. Ecologically, this sustained greening trend indicates a significant recovery of the regional vegetation cover, which translates to enhanced soil retention capacity and improved terrestrial carbon sequestration in the basin.

**Figure 3b** shows the multi-year average NDVI and NDVI slopes for each sub-basin. The average NDVI for the UB_YRB (upper basin), MB_YRB (middle basin), and LB_YRB (lower basin) increased from 2001 to 2020, with values of 0.509, 0.640, and 0.762, respectively. In the UB_YRB, there were alpine vegetation (100%), deserts (100%), alpine meadows (97%), swamps (94%), and grasslands (73%). In the MB_YRB and LB_YRB, there were tussock lands (96%), broad-leaved forest (84%), cultivated vegetation (80%), and coniferous forest (67%). The spatial distribution of different vegetation types directly influenced the NDVI differences among the three sub-basins of the YRB.

Compared with the UB_YRB and LB_YRB, the MB_YRB showed the highest green rate (*slope* = 0.0074 per decade, *p* < 0.05). On the one hand, the Grain for Green projects on the Loess Plateau altered the land use structure and converted plenty of arable slope land into grassland and forestland (Gu et al., 2021b). On the other hand, more than 70% cultivated vegetation had been established in the MB_YRB. Favorable hydrothermal conditions further promoted vegetation growth in the MB_YRB. In addition, the average NDVI of UB_YRB, MB_YRB, and LB_YRB increased by 8%, 12%, and 2% during the period from 2011 to 2020 compared with those from 2001 to 2010. However, the NDVI showed a decreasing trend in UB_YRB from 2011 to 2020, which differed from the steady rising trend in MB_YRB and LB_YRB.

#### 4.1.2 Spatial changes of multi-year NDVI

**Figure 4:**
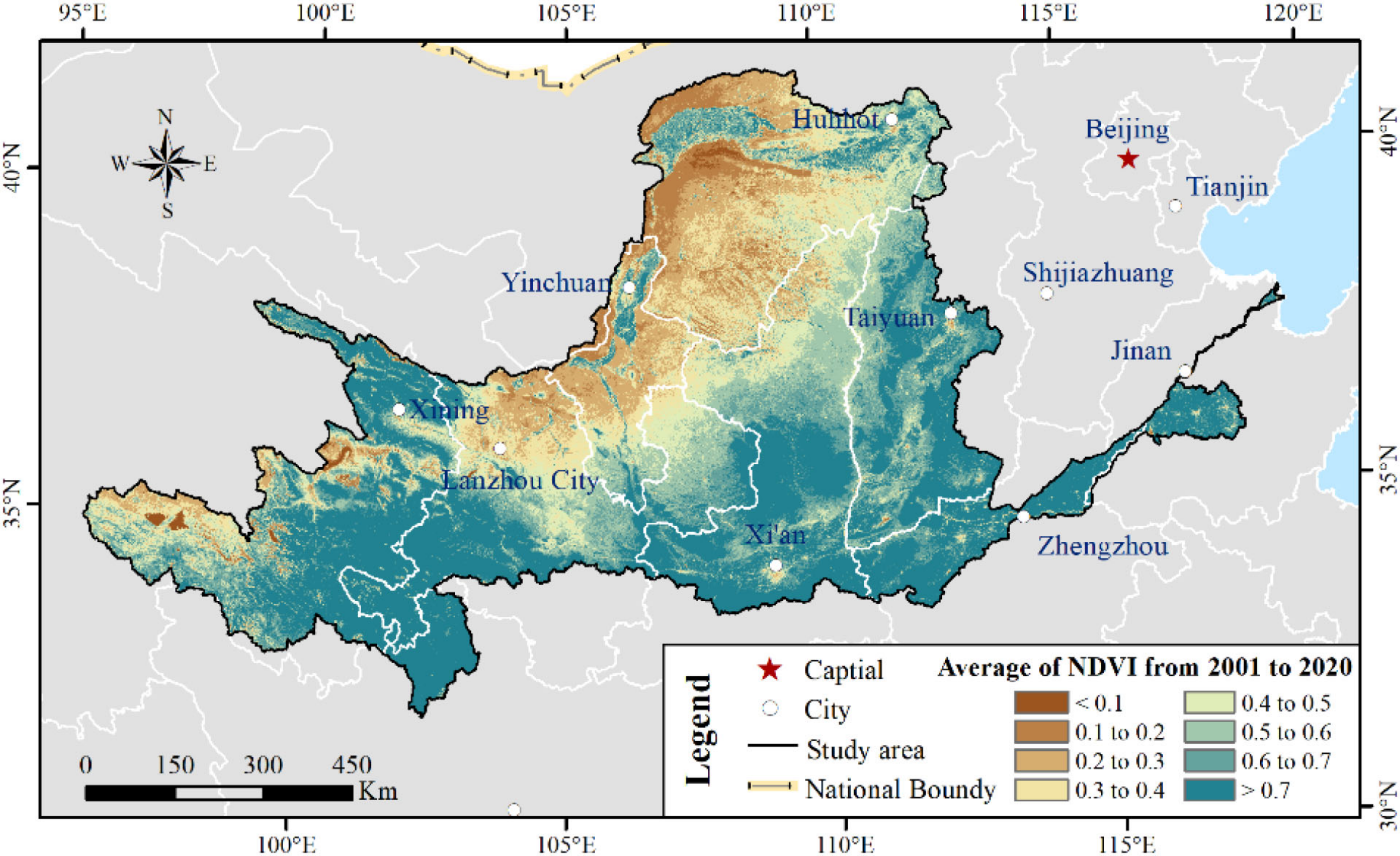
Average NDVI from 2001 to 2020. (NDVI less than 0.1 means non-vegetation areas.).

**Figure 5:**
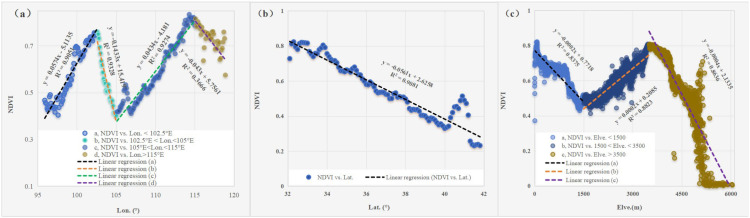
Zonal distribution characteristics between average NDVI and longitude **(a)**, latitude **(b)**, and elevation **(c)**.

Based on the 20-year NDVI dataset, the spatial distribution of the average NDVI calculated by ArcGIS10.2 is given in **Figure 4**. The average NDVI varied between 0.1 and 0.95, with an average value of 0.57, during the period from 2001 to 2020 in the YRB. The NDVI was universally less than 0.3 in the UB_YRB, accounting for approximately 15% of the area, where barren land or low vegetation coverage was concentrated. Vegetation growth was restricted due to a lack of enough water and/or low temperature (Yuan et al., 2021). However, the NDVI was greater than 0.7 which was mostly distributed in the partition of nearly the whole LB_YRB (more than 80% distribution area), MB_YRB (approximately 38% distribution area), and the Yellow River source regions (nearly 30% distribution area). Vegetation growth varied with the various latitude and longitude (**Figure 5**). With the latitude decreasing, NDVI failed to enhance. As we all know, when the latitude decreases, the temperature will drop. This may explain why NDVI was greater than 0.7 under 35° N.

With the spatial pattern of average NDVI, we illustrate the relationship between NDVI and latitude, longitude, and elevation in **Figure 5**. Between 95° E and 102.5° E and between 105° E and 115° E, the NDVI increased by 0.057 per degree and 0.043 per degree in longitude. However, a significantly decreasing rate of NDVI between 102.5°E and 105°E and between 115°E and 120°E was approximately 0.143 and 0.043 per degree, respectively (**Figure 5a**). We observed a significantly decreasing rate of NDVI as the latitude increased, defining a green rate of 0.056 per degree in latitude (**Figure 5b**). As the elevation increased, ranging between 1,500 and 3,500 m, the NDVI enhanced by 0.02 per 100 m. When the elevation was more than 3,500 m or less than 1,500 m, NDVI reduced by 0.02 per 100 m or 0.04 per 100 m (**Figure 5c**).

#### 4.1.3 Spatial pattern of NDVI trend change

**Figure 6:**
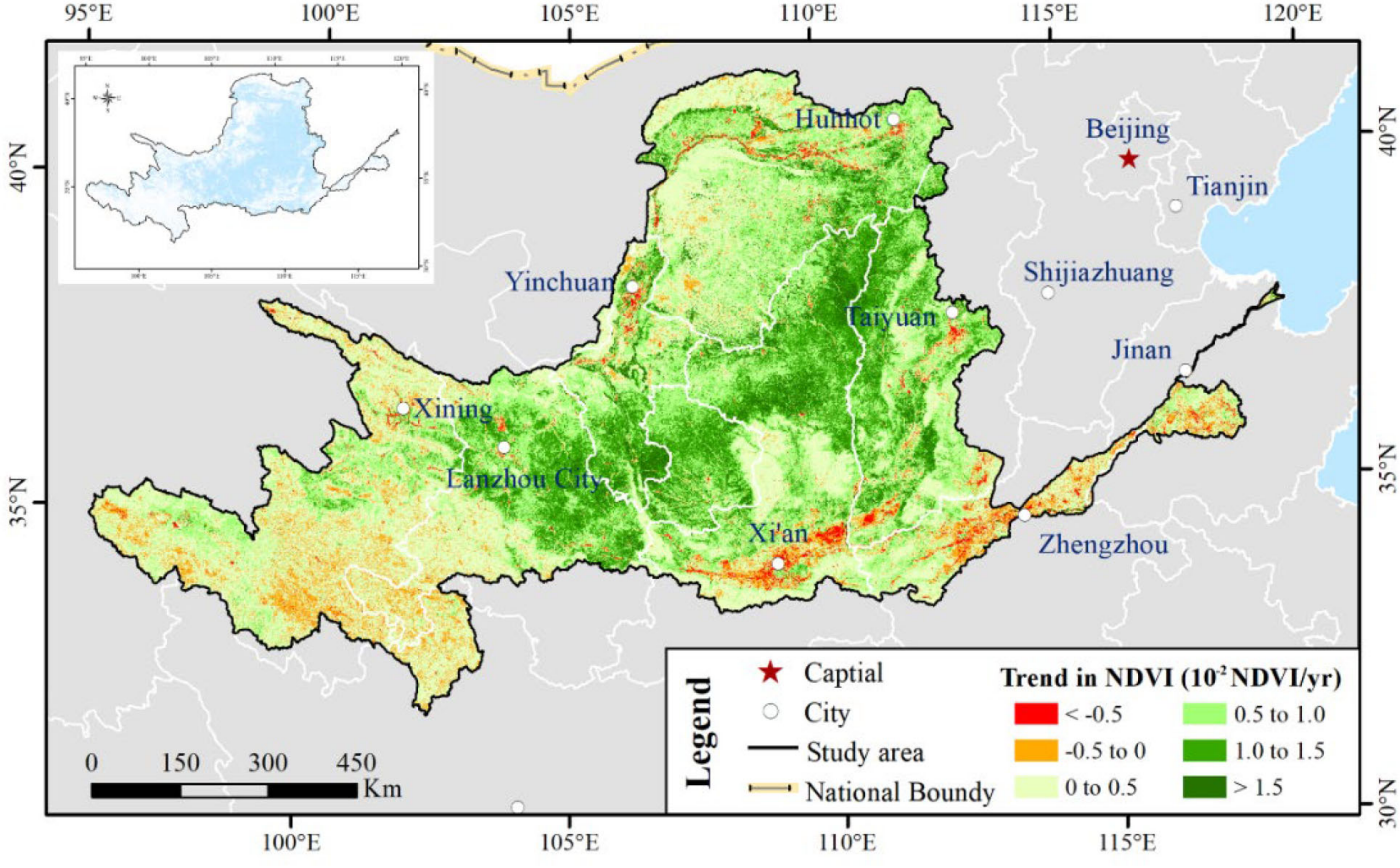
Spatial trend pattern of annual NDVI. The small inset figure reveal the significance levels of the spatial trend pattern. The trend variation of annual NDVI was significant in the blue region at 95% significance level.

With the 20-year (2001 to 2020) grid annual NDVI, we applied linear regression to calculate the spatial trend pattern of annual NDVI over YRB and their magnitudes (10–^2^ NDVI per decade) in **Figure 6**. The NDVI increased in more than 90% of the total areas in the YRB, which was significant at *p <*0.05, accounting for approximately 50% and primarily located in the MB_YRB. During the 21st century, vegetation showed greening in more than 94% of the YRB regions (Feng et al., 2021). In contrast, approximately 10% of the areas of the YRB had a negative trend of NDVI. The regions with a sharply decreasing trend (slope < -0.05 per decade) were discovered piecemeal in the LB_YRB, the southeast of MB_YRB (such as Yiluo River Basin) and in parts of the Loess Plateau of the UB_YRB. More than 8% slight decrease (-0.05 per decade < slope < 0) occurred in the Yellow River source regions and LB_YRB, especially in southeastern Qinghai.

**Figure 7:**
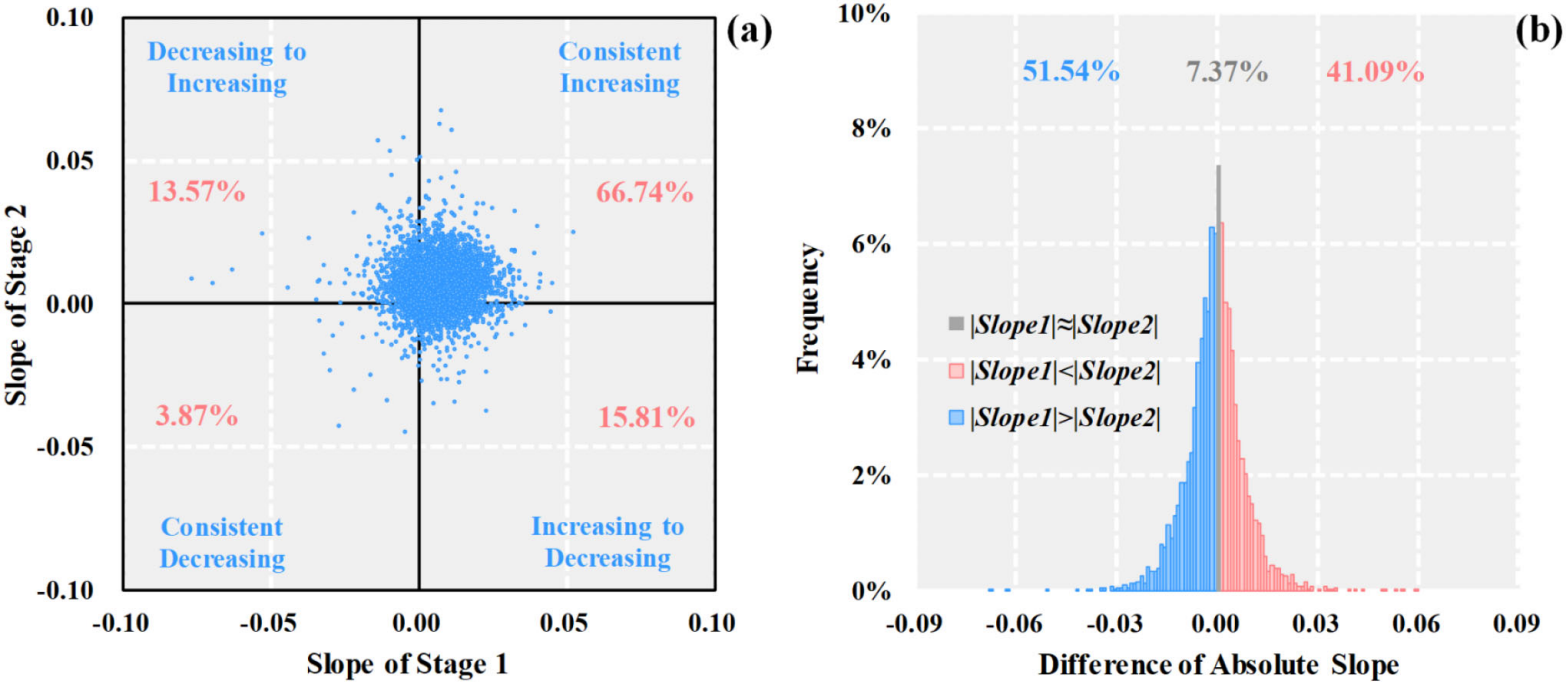
Scatter plot for NDVI slope **(a)** and histogram for difference of NDVI absolute slope value **(b)**. The percentages express the proportion of pixel amount in the corresponding quadrant.

We used ArcGIS 10.2 to create 5,755 (5 km) random points from a total of 828,523 pixels in YRB, of which each pixel was 1 km × 1 km, and we divided the study period into two stages: stage 1 was from 2001 to 2010 and stage 2 was from 2011 to 2020. **Figure 7** compared the NDVI slopes of 5,755 random points for stages 1 and 2. Approximately 67% of all pixels had a consistent increasing inclination in both stages, and approximately 4% of all pixels showed a consistently decreasing tendency in stages 1 and 2. The proportion of NDVI decreasing-to-increasing tendency and increasing-to-decreasing tendency accounted for 14% and 16% of all pixels, respectively (**Figure 7a**). On the other hand, in **Figure 7b**, more than 50% pixels emerged with a higher change slope in stage 1, with a higher absolute slope value than stage 2. In contrast, we detected at the rate of 41% pixels with a lower change slope in stage 1, which implied a lower absolute slope value than stage 2. These performances revealed that vegetation experienced faster or slower greening or degradation in both stages. These results were consistent with existing research (Yuan et al., 2021).

### 4.2 Relationship between NDVI and major hydrothermal conditions

#### 4.2.1 Spatial–temporal characteristics of hydrothermal conditions

**Figure 8:**
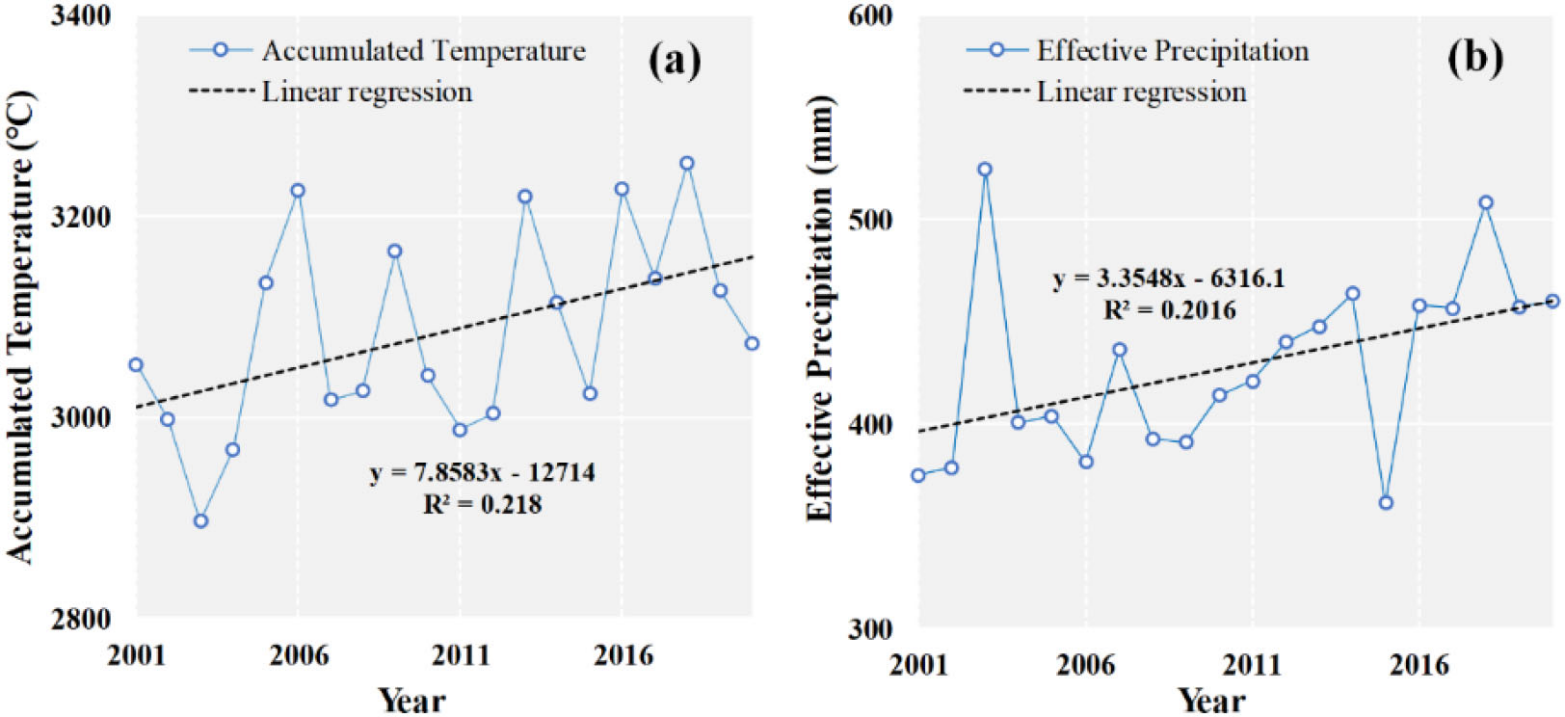
Inter-annual variation of accumulated temperature (ACTE) **(a)** and effective precipitation (EFPR) **(b)** in the YRB during the period from 2001 to 2020, showing an overall warming and wetting trend with interannual fluctuations.

**Figure 8** illustrates the trend variations of ACTE and EFPR over the entire YRB. The annual ACTE increased from 2,897°C in 2003 to 3,254°C in 2018. An ACTE presented an increasing trend with some fluctuation and reach to 3,074°C in 2020, prefiguring a decreasing-to-increasing shift (**Figure 8a**). A consistently increasing trend in annual average EFPR was obtained from 2001 to 2020 as shown in **Figure 8b**. The EFPR trend magnitude was 3.35 mm per year during the period from 2001 to 2020. The difference between the maximum (524 mm, 2003) and minimum (361 mm, 2015) EFPR was 163 mm. Overall, climate had become wetter and warmer since 2000 over YRB. The wetter and warmer climate might have led to the greener vegetation (Yuan et al., 2021).

**Figure 9:**
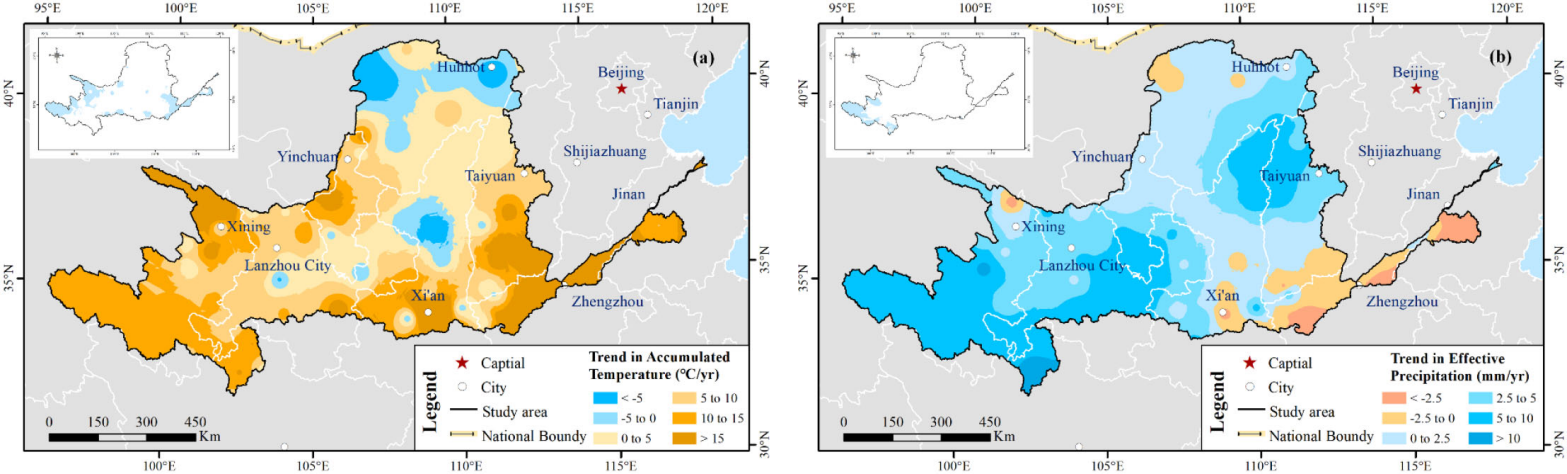
Spatial patterns of trends in annual accumulated temperature (ACTE) **(a)** and effective precipitation (EFPR) **(b)** across the Yellow River Basin (YRB). Insets indicate the significance levels of trend patterns, with blue areas representing statistically significant trends at the 95% confidence level. The figure highlights the contrast between widespread warming–wetting trends and localized warming–drying regions.

**Figure 9** shows the spatial trend distribution of ACTE (**Figure 9a**) and EFPR (**Figure 9b**). The positive changes of ACTE (approximately 87% of the regions) and EFPR (approximately 89% of the regions) distributed nearly in the most area of the YRB, of which the variation rate was 7.9 °C per decade and 3.4 mm per year, respectively. However, for the ACTE, its proportion of areas with a significantly positive trend (*p* < 0.05) areas was approximately 20%, and for the EFPR, only approximately 8.5% of the regions have a positive variation that was significant (*p* < 0.05). The areas with a negative trend of ACTE and EFPR accounted for 13% and 11% of the total, respectively. However, their trend changes failed the test of significance. Regions with decreasing ACTE trend were mainly located in the northern part of YRB and partially of MB_YRB. The major decreasing EFPR trend occurred in nearly the whole LB_YRB and locally of MB_YRB (such as Yiluo River Basin). Regions that exhibited the warming–drying tendency include the LB_YRB and parts of MB_YRB (such as Yiluo River Basin).

#### 4.2.2 NDVI changes related to ACTE and EFPR

**Figure 10:**
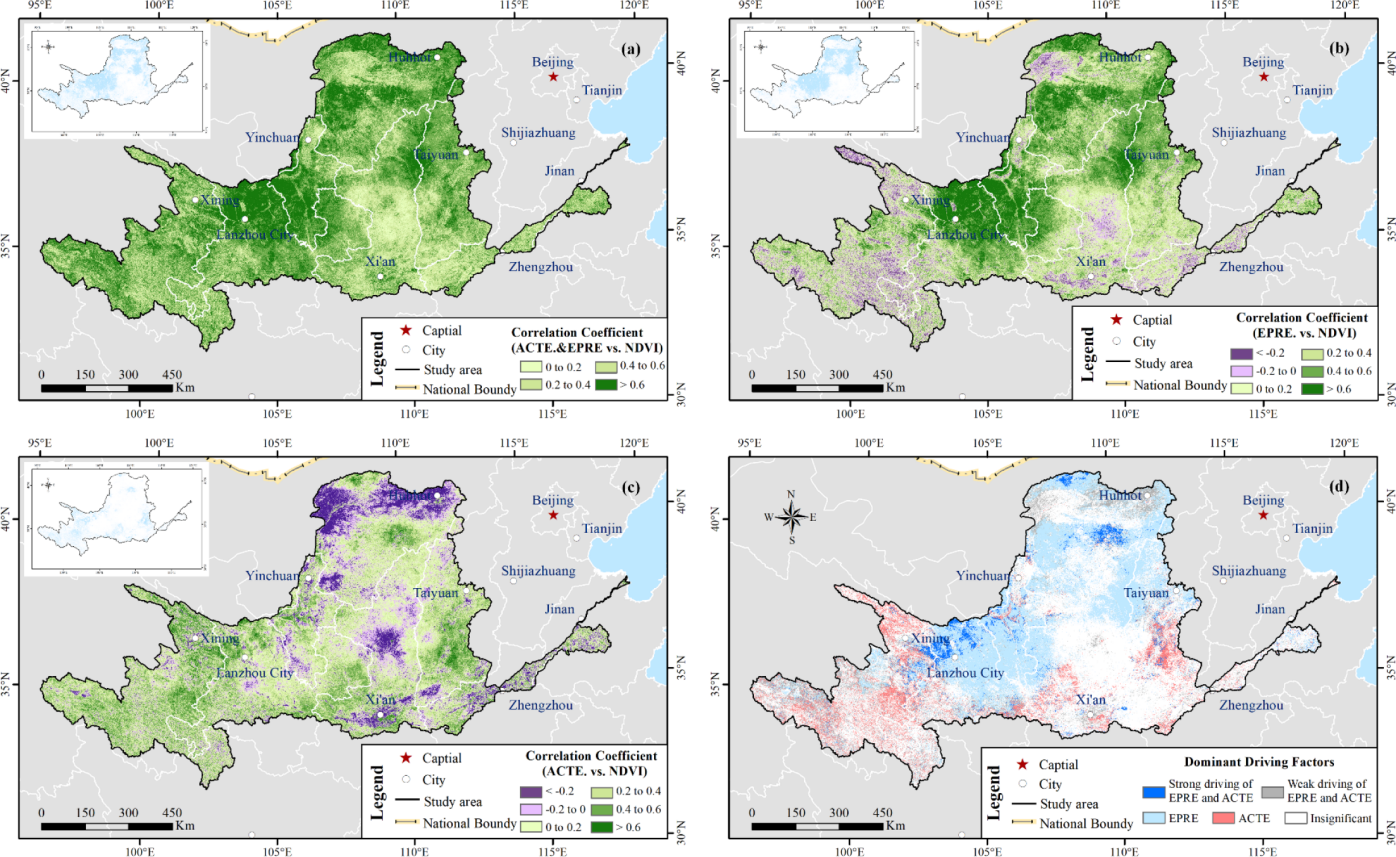
Spatial patterns of correlation coefficients, significance levels, and dominant climatic driving factors affecting NDVI in the vegetated Yellow River Basin (YRB) from 2001 to 2020. **(a)** Multiple correlation coefficients between NDVI, accumulated temperature (ACTE), and effective precipitation (EFPR), with blue inset areas indicating statistically significant correlations (*p* < 0.05). **(b)** Partial correlation coefficients between NDVI and EFPR and their significance levels. **(c)** Partial correlation coefficients between NDVI and ACTE and their significance levels. **(d)** Dominant climatic driving factors controlling NDVI variations, highlighting regions mainly influenced by EFPR, ACTE, or their combined effects.

We applied multiple linear regression to investigate the response of NDVI to ACTE and EFPR. **Figure 10a** shows their multiple correlation coefficients and significance level at 95% confidence level. Approximately 34% of the regions with high multiple correlation coefficients passed the significance test, and nearly 22% were located in the UB_YRB, especially in southeastern Gansu, southwestern Ningxia, the Ordos Plateau, and Hetao Plain of Inner Mongolia. A total of 11% of these regions were concentrated in the MB_YRB, especially in the Loess Plateau of southern Weihe River (the largest branch of the Yellow river) and Lvliang mountain areas. These results indicate that the higher climate contributions contributed to vegetation growth in the abovementioned areas.

**Figures 10b,c** illustrate the partial correlation between NDVI and EFPR and ACTE. Approximately 70% of the pixels of NDVI was positively correlated with EFPR, of which approximately 15% exhibited significant positive correlations (*p* < 0.05). The pixels with a significant positive correlation were generally consistent with the general regions characterized by significant multiple correlation. More than 30% pixels had a negative correlation instead, all of which failed to pass the significance test.

**Figure 10d** shows the spatial pattern of prominent climatic driving factors affecting NDVI in vegetated YRB from 2001 to 2020. The proportion was more than 45% of the vegetated YRB, which significantly (*p* < 0.05) affected NDVI by EFPR and/or ACTE. Regions dominated by EFPR accounted for 27% and were relatively centralized in the Hobq Desert of eastern Ho-lan Mountains and the Loess Plateau of southern Weihe river, while regions predominated by ACTE were approximately 8% and were generally distributed, the Yellow River resource and nearby Weihe River, Fenhe River, and Jinghe River. Furthermore, 10% of the total significant regions were dominated by the EFPR and ACTE and mainly distributed in part of the Hobq Desert, the section of Huangshui River Basin, and the northern of YRB.

### 4.3 Relationship between NDVI and human activities

#### 4.3.1 NDVI characteristics related to night light

**Figure 11:**
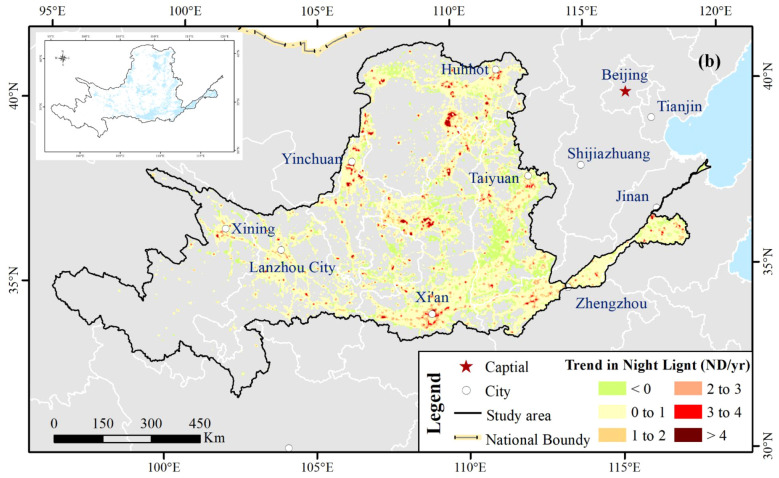
Spatial trend distribution of annual night light (NL). The small inset figure reveal the significance levels of the spatial trend pattern. The trend variation of annual NL was significant in the blue region at 95% significance level.

**Figure 12:**
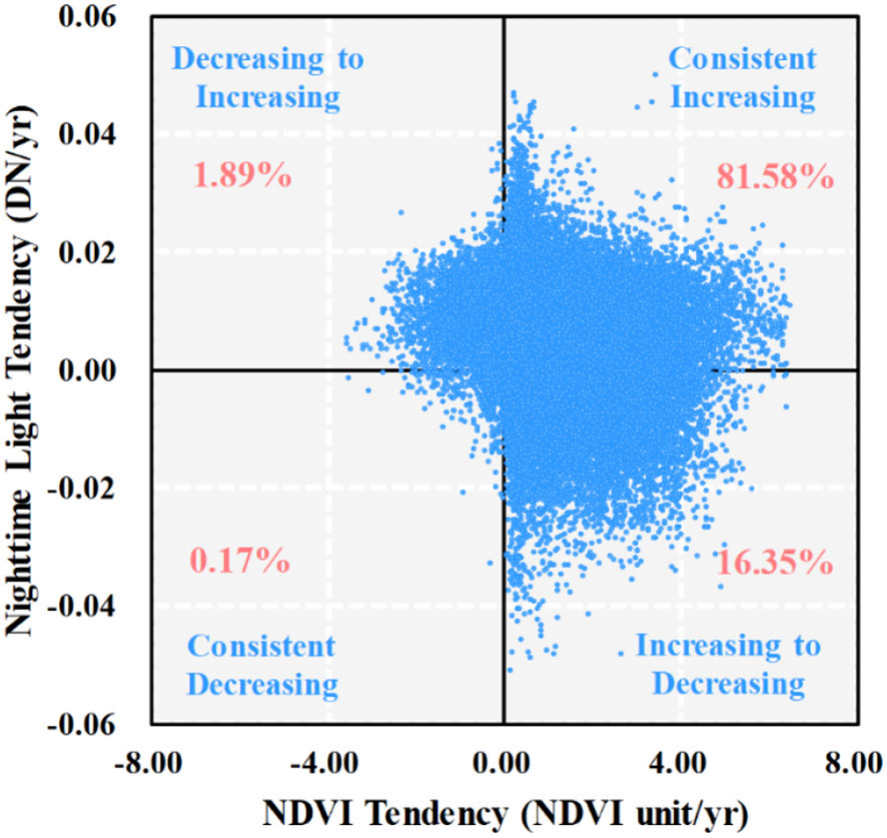
Scatter plot for NDVI and night light (NL) tendency at 95% significance level. Quadrants indicate different combinations of NDVI and NL changes, illustrating areas where vegetation greening or browning is associated with urbanization intensity.

**Figure 11** shows the spatial variation of the annual NL trend. For NL, its proportion of areas with significantly positive trends (*p* < 0.05) was more than 20%. The research showed the ongoing urbanization developed in China since 2001, especially in the Huang-Huai-Hai Plain (Yuan et al., 2021). We extracted the significant NL tendency, converted the raster to points and then extracted the significant NDVI trend value to points. **Figure 12** illustrates that approximately 82% of the scattered points increased both NDVI and NL in quadrant I, which implied that a proportion of vegetation was enhanced during the urbanization over YRB. However, the faster vegetation greening was performed in regions of slower urbanization (Yuan et al., 2021). Approximately 18% of the scattered points suggested that urbanization affected negatively the vegetation NDVI variation, which performed decreasing to increasing or increasing to decreasing in quadrants II and IV, respectively. Only nearly 0.2% of the scattered points had both decreased NDVI and NL in quadrant III.

The spatial trend distribution of annual NL demonstrates rapid urbanization in the YRB since 2000. The building land over YRB had expanded from approximately 1.7 × 10^4^ km^2^ in 2000 to 2.8 × 10^4^ in 2020, of which the growth rate rose nearly by 65% during 20 years. Compared with the land pattern in 2000, the proportion of cultivated land, forestland, and grassland shrank by 6.4%, 1.1%, and 1.4%, respectively, which were transformed to construction land and generated approximately 54%, 5%, and 21% of its formation in 2020. Accelerated urbanization occupied large areas of arable land. The LB_YRB was identified as the concentration region of urbanization, of which the transformed portion was nearly 30%.

#### 4.3.2 NDVI variations related to forestlands and croplands

Compared with the period before 2010, the forest area proportion increased only 8.6% by expanding natural forests and afforestation after 2010, which showed that it had a comparable and stable forest area to that in the YRB. The “Three North” Shelterbelt Development Program (TNSDP), the Nature Forest Conservation Program (NFCP), and the Grain to Green Program (GTGP) increased the greenness tendency (Zhang et al., 2016) in the YRB. During the period from 2011 to 2020, the average accumulated afforested area was 231.16 × 10–^4^ hm, accounting for approximately 22.4% of the current forest area (**Table 4**).

**Table 4:** Changes in the forest and accumulated afforested forest from 2004 to 2020.

Year	Forest area (10^4^ hm)	Forest cover rate (%)	Accumulated afforested area (10^4^ hm)
2011–2020	800.46	21.25	231.16
2004–2010	731.88	19.08	179.46
Change (2004–2020)	68.57 (8.6%)	2.17	51.69 (22.4%)

**Figure 13:**
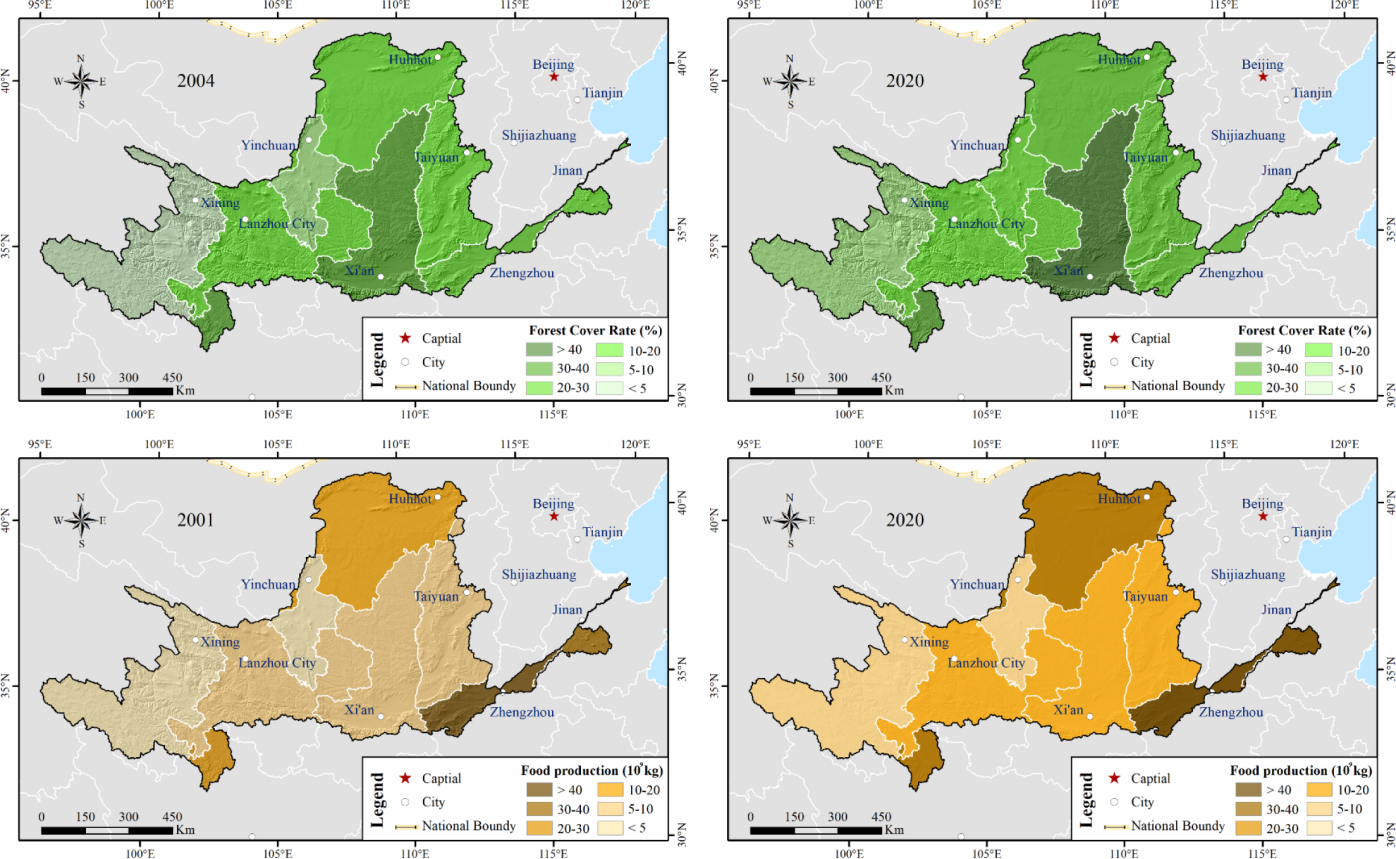
Forest cover rate in 2004 and 2020 and the total food production in 2001 and 2020.

In the last 10 years, the harvested area remained relatively stable, increasing by only 5.5% compared with that before 2010 (**Table 5**). However, the total food production increased by approximately 25%, which could be caused by the utilization of irrigation and fertilizer. The effective irrigation area had expanded by 16.4%, and the volume of fertilizer used had risen by 24.7% after 2010. **Figure 13** illustrates the spatial pattern of the forest cover rate and food production. In the Shandong and Henan provinces, food production enlarged significantly. Meantime, the Shanxi province developed massive forestation from 2011 to 2020. These changes likely contributed to vegetation greening, as reflected in the NDVI trends shown in **Figure 6**.

**Table 5:** Changes in the major croplands from 2004 to 2020.

Year	Harvested area (10^4^ hm)	Effective irrigation area (10^4^ hm)	Total food production (10^4^t)	Fertilizer use amount (10^4^t)
2011-2020	632.14	232.76	247.72	22.97
2001/2-2010	597.06	213.70	185.94	19.40
(2001/2-2020)	35.08 (5.5%)	19.05 (8.2%)	61.78 (24.9%)	3.57 (15.5%)

### 4.4 Effects of NDVI responses to climate changes and human activities in the vegetation variations

#### 4.4.1 Spatial pattern of the various driving forces tendency

**Figure 14:**
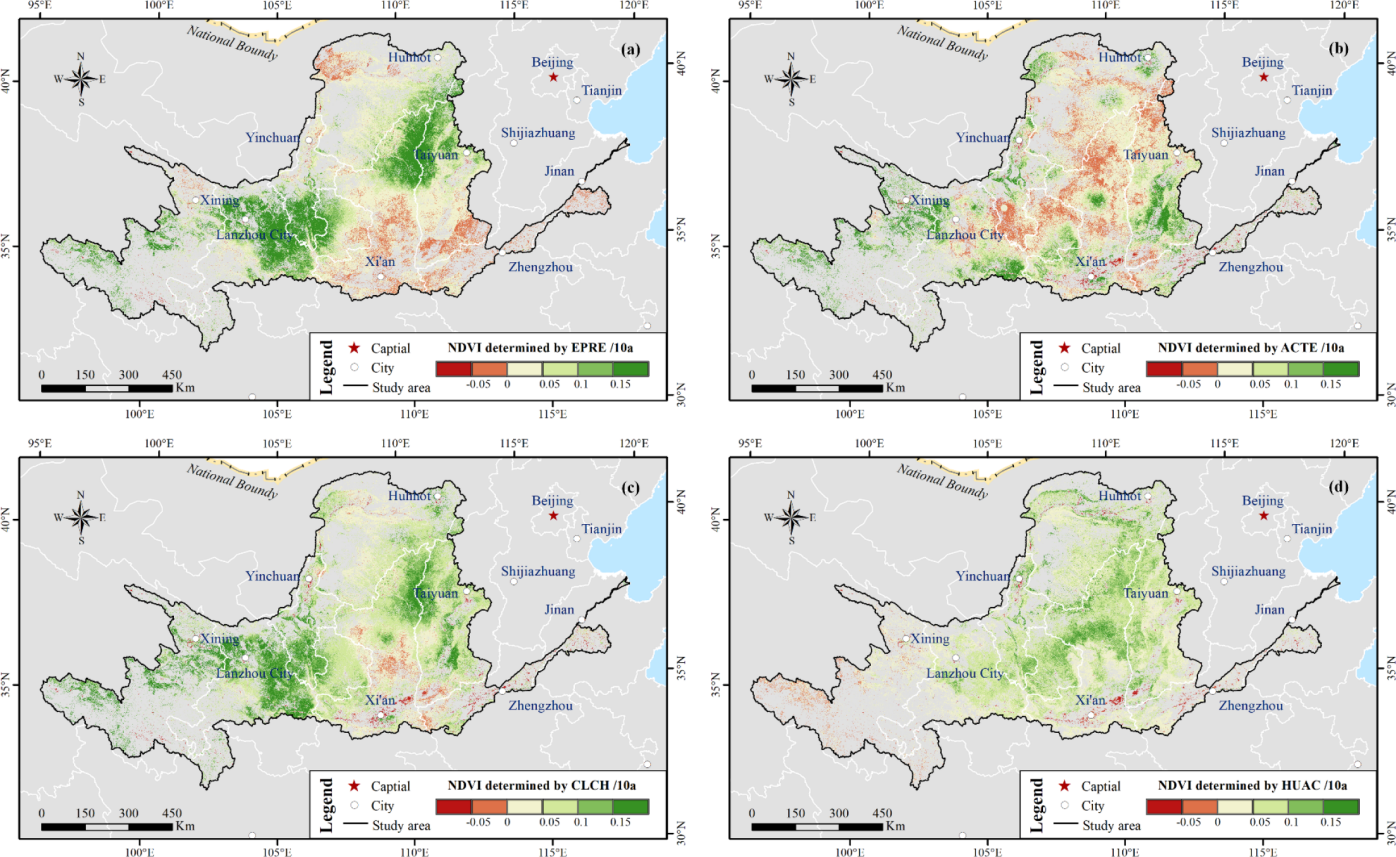
Spatial patterns of contributions from climatic and anthropogenic factors to NDVI variations in the Yellow River Basin (YRB) from 2001 to 2020. **(a)** Contribution of effective precipitation (EFPR), **(b)** contribution of accumulated temperature (ACTE), **(c)** contribution of climatic factors (CLCH), and **(d)** contribution of human activities (HUAC). The figure highlights the dominant role of human activities in most regions and the contrasting influence of climatic factors in specific sub-regions.

The contribution pattern of climate forces and human activities differed from 2001 to 2020 throughout YRB. In the whole YRB, the average contributions of the EFPR, ACTE, and CLCH were 0.012, 0.007, and 0.019 per decade, of which the homologous contribution rates were approximately 22%, 13% and 35%, respectively. The EFPR was the restrictive factor for the NDVI dynamics. **Figure 14** shows the spatial distribution of the major driver contribution that affected the NDVI variations. For the climate changes (**Figures 14a-c**), in the areas where NDVI showed a significant tendency (*p* < 0.05), CLCH increased (CLCH > 0.01 per decade) in approximately 72% of the regions where climate factors positively affected the NDVI. The area with EFPR and ACTE (above 0.01 per decade) covered 49% and 35%, respectively. The regions were concentrated in the Weihe–Jinghe regions over Chinese Loess Plateau and in the Shaanxi–Shanxi section on the Main Yellow River, where NDVI was determined by the EFPR (**Figure 14a**), which coincided with the CLCH effects (**Figure 14c**), and the areas revealed a discrete geographical distribution where the ACTE increased (**Figure 14**)).

The average contribution of the HUAC was 0.036 per decade, accounting for approximately 65% of the total NDVI variation (**Figure 14d**), indicating that human activities played a dominant role in vegetation changes. In other words, the area where human factors affected the NDVI positively (HUAC > 0.04 per decade) covered approximately 70%. These regions were concentrated in the Chinese Loess Plateau and Lvliangshan Mountain regions over the MB_YRB, whereas only in the Xi’an, Zhengzhou, Xining cities, and parts of Yellow River sources exhibited NDVI browning by human activities.

#### 4.4.2 Contributions of climate changes and human activities on the NDVI dynamics

**Figure 15:**
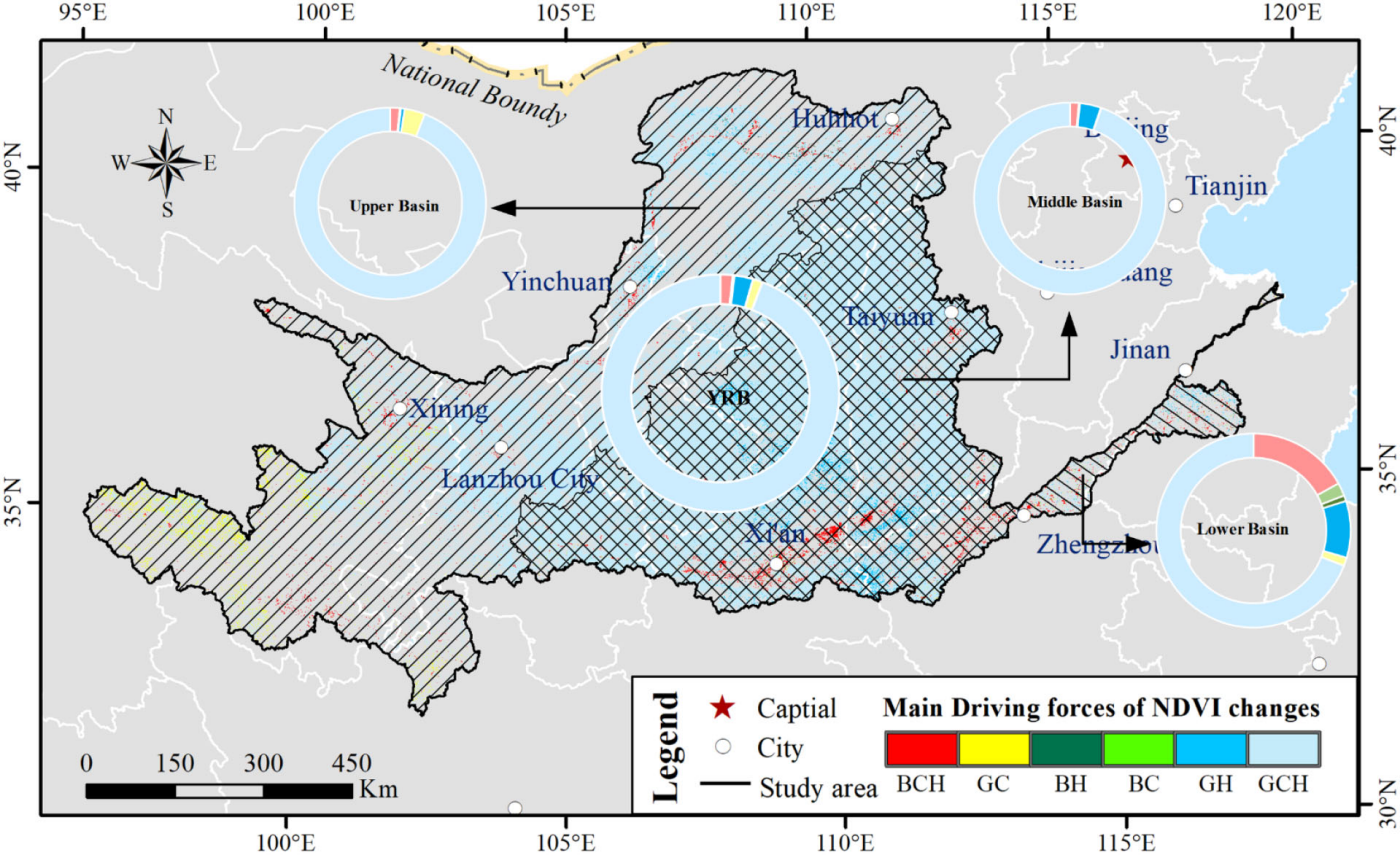
Spatial distribution of dominant driving factors controlling NDVI variations in different sub-basins of the Yellow River Basin (YRB) where NDVI trends are statistically significant (*p* < 0.05) since 2001. The figure distinguishes regions dominated by climatic factors, human activities, or their combined effects.

Climate changes and human activities jointly influenced the NDVI dynamics over YRB. We extracted the main driving forces of NDVI variations at the pixel level that were statistically significant (*p* < 0.05) (**Figure 15**). Vegetation showed greening caused by climate change and human activities. The proportion of enhancing NDVI was approximately 94%. The proportion of browning vegetation was approximately 2% instead because of climate change and human activities. The NDVI diminished by both driving forces was mainly distributed in the urban regions, such as Xi’an, Zhengzhou and Xi’ning cities. The NDVI increases driven only by climate factors or human activities was approximately 1% and 3%. These areas were distributed in the UB_YRB and LB_YRB.

**Figure 16:**
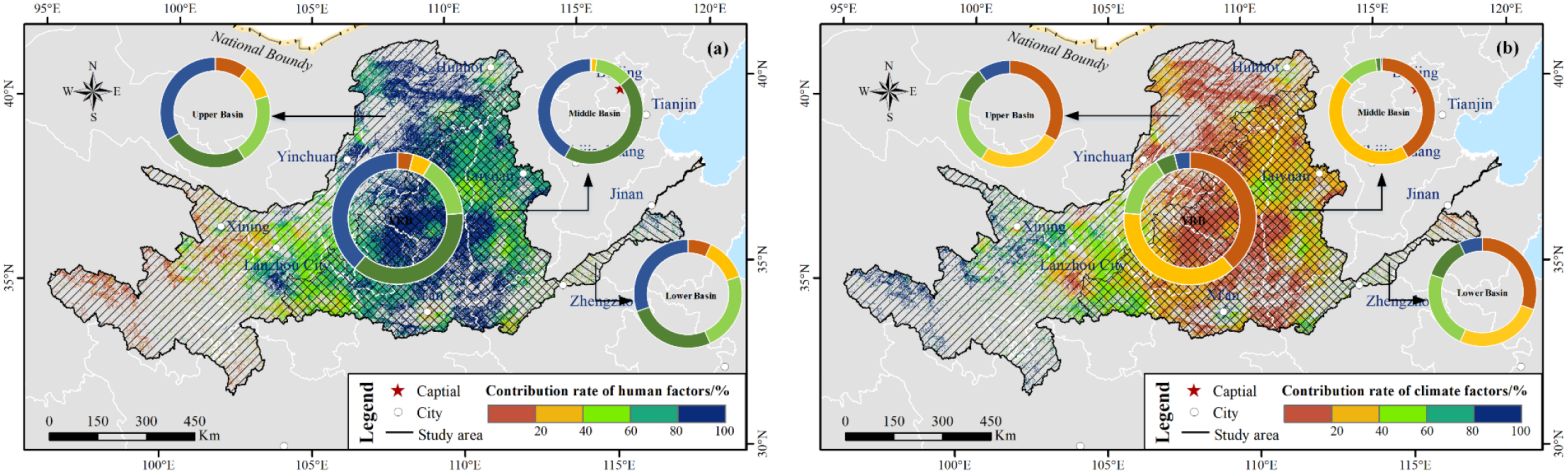
Contributions of the anthropogenic factors **(a)** and climatic factors **(b)** to NDVI variations in the various basins where NDVI performed significantly at 95% significance level from 2001 to 2020.

In the areas where NDVI showed significant trends (*p* < 0.05), regions in the Yellow River source area accounted for less than 4%, where the human contribution rate was below 20% (**Figure 16a**). The anthropogenic factors contributed nearly four out of five, and approximately 40% regions were distributed in the MB_YRB. **Figure 16b** shows that the contribution rate of the climatic factors from 0% to 40% mainly concentrated in the MB_YRB, which accounted for approximately 76%. Climatic factors were restrictive drivers of the NDVI variations in the regions (less than 10%), where the contribution rate was more than 60%. These regions were mainly distributed in the Yellow River source region.

### 4.5 Discussion

#### 4.5.1 Comparison of the results with EVI

**Figure 17:**
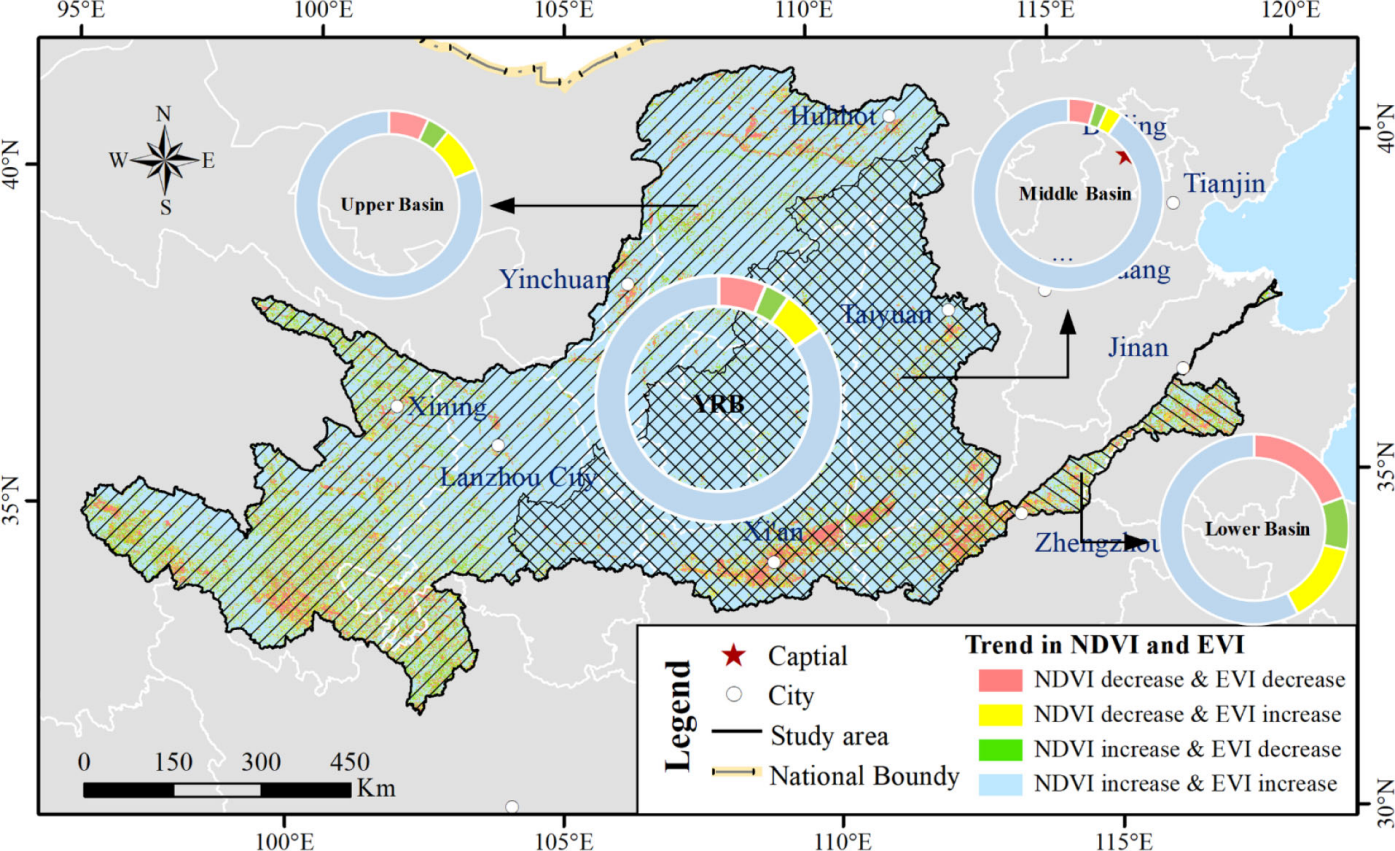
Spatial variation tendency between NDVI and EVI over the basins.

Besides NDVI, EVI (enhanced vegetation index) is also applied for vegetation estimation (Shammi and Meng, 2021; Yan et al., 2018; Zhu et al., 2019). We analyzed EVI data for the corresponding period as derived from the NASA MODIS Global Vegetation Index product (https://www.nasa.gov/) by the same methods mentioned in Section 3. **Table 6** shows that changes of the EVI and NDVI exhibited similar proportions in the sub/whole basins. **Figure 17** illustrates the temporal pattern of the EVI and NDVI change proportions. Changes of the EVI and NDVI were accord in more than 90% area (region colored by blue and red) and mainly disaccord in the UB_YRB, especially in the Yellow River source region where the vegetation is sparse. The vegetation growth was obviously worse than that in the MB_YRB and LB_YRB, which coincided with the research performed by Tian et al. (2021). From the land use type distribution (**Figure 2**), it was clear that more than 85% of the barren land are concentrated in the UB_YRB, and the major vegetations were alpine vegetation (such as *Arenaria bryophylla*, *Androsace tapete* Maxim, and *Saussurea japonica* sparse vegetation, etc.), shrublands (such as *Amygdalus mongolica* (Maxim.) Ricker, *Potentilla fruticosa* L., *Salix oritrepha* Schneid, etc.), meadow, and marsh in the UB_YRB, and both EVI and NDVI trends indicate that vegetation browning mainly occurred in the LB_YRB, where the average NDVI was the highest and with the fastest urbanization than that in the MB_YRB and UB_YRB.

**Table 6:** Changes in EVI and NDVI over sub-basins and whole basin.

Vegetation index	Slope > 0 (%)		Slope < 0 (%)	
EVI	UB_YRB	48.04	UB_YRB	5.86
	MB_YRB	40.34	MB_YRB	2.93
	LB_YRB	2.02	LB_YRB	0.81
	YRB	90.41	YRB	9.59
NDVI	UB_YRB	45.81	UB_YRB	8.09
	MB_YRB	40.12	MB_YRB	3.15
	LB_YRB	1.88	LB_YRB	0.94
	YRB	87.81	YRB	12.19

UB_YRB, upper basin of YRB; MB_YRB; middle basin of YRB; LB_YRB, lower basin of YRB.

#### 4.5.2 Response of the NDVI to climatic factors

Climatic forces are the most important drivers of vegetation change (Kong et al., 2020; Li et al., 2021). Most studies have identified precipitation and temperature as the major climatic factors that affect the vegetation temporal growth and spatial distribution (Kong et al., 2020; Yin et al., 2020; Yuan et al., 2021). However, the vegetation growth exhibits a distinct time lag in response to precipitation and temperature (Li et al., 2021; Zhao et al., 2020). Considering the cumulative effects of precipitation and temperature, we incorporated the impacts of the EFPR and ACTE on vegetation into account, which were ignored in previous studies. The EFPR is defined as the amount of rainfall that could infiltrate into the soil and be useful directly and/or indirectly for crop production during the crop growing cycle (Akhtar and Athar, 2020; Ali and Mubarak, 2017; Marek et al., 2016). As shown in **Figure 9**, nearly 77% of the regions exhibited wetting and warming trends over YRB since 2000. The correlation analysis identified EFPR and ACTE as the dominant climatic factors affecting NDVI (**Table 2**).

Except for the Yellow River source region, the Hetao irrigation regions, and the parts of the southeastern basin, the EFPR was the primary limiting factor contributing to NDVI dynamics. In arid and semi-arid areas, water changes affect vegetation productivity and resilience (Liu et al., 2018b). Rising in precipitation and increasing in vegetation cover tends further provided that water availability was a restrictive factor for vegetation growth (Peng et al., 2019). In some local areas, reductions in vegetation were found where the precipitation decreased (Yuan et al., 2021). In temperate regions, dry land vegetation was constrained by hydrothermal conditions (Peng et al., 2019). In the Yellow River source regions, compared with the EFPR, the ACTE made a greater positive contribution for vegetation variations. These regions were high elevation, low temperature, and the harsh alpine environment. The average ACTE in these regions was approximately 1,500°C. In alpine areas, 2,000°C ACTE was needed to meet the vegetation growth during the whole growth season of vegetation (Gong et al., 2018). The harsh climatic conditions were not favorable for vegetation growth (Yuan et al., 2021). Specifically, the dominance of climatic factors in these alpine source regions is fundamentally driven by physiological constraints. Low temperature serves as the primary limiting factor for enzymatic activity and photosynthesis in high-elevation ecosystems (Zhou et al., 2021). Consequently, the observed warming trend (increased ACTE) directly relieves this thermal constraint, extending the phenological growing season and enhancing the net primary productivity (Liu et al., 2018c; PIAO et al., 2006).

#### 4.5.3 Response of the NDVI to anthropogenic factors

Afforestation projects have accelerated vegetation restoration (Li et al., 2021; Yin et al., 2020), especially the Three North Shelterbelt Development Program, an afforestation program for Taihang Mountain and a shelterbelt program for the middle reaches of Yellow river. The Grain for Green Program in the Loess Plateau changed the spatial–temporal vegetation pattern and increased the NDVI variation trend (0.057 per decade) during 2001 to 2015 (Li et al., 2021). From 2001 to 2020, the tendency of NDVI changes was 0.069 per decade in our study. This finding was consistent with existent studies that revealed the greening pattern in China (Chen et al., 2019; Yuan et al., 2021).

**Figure 18:**
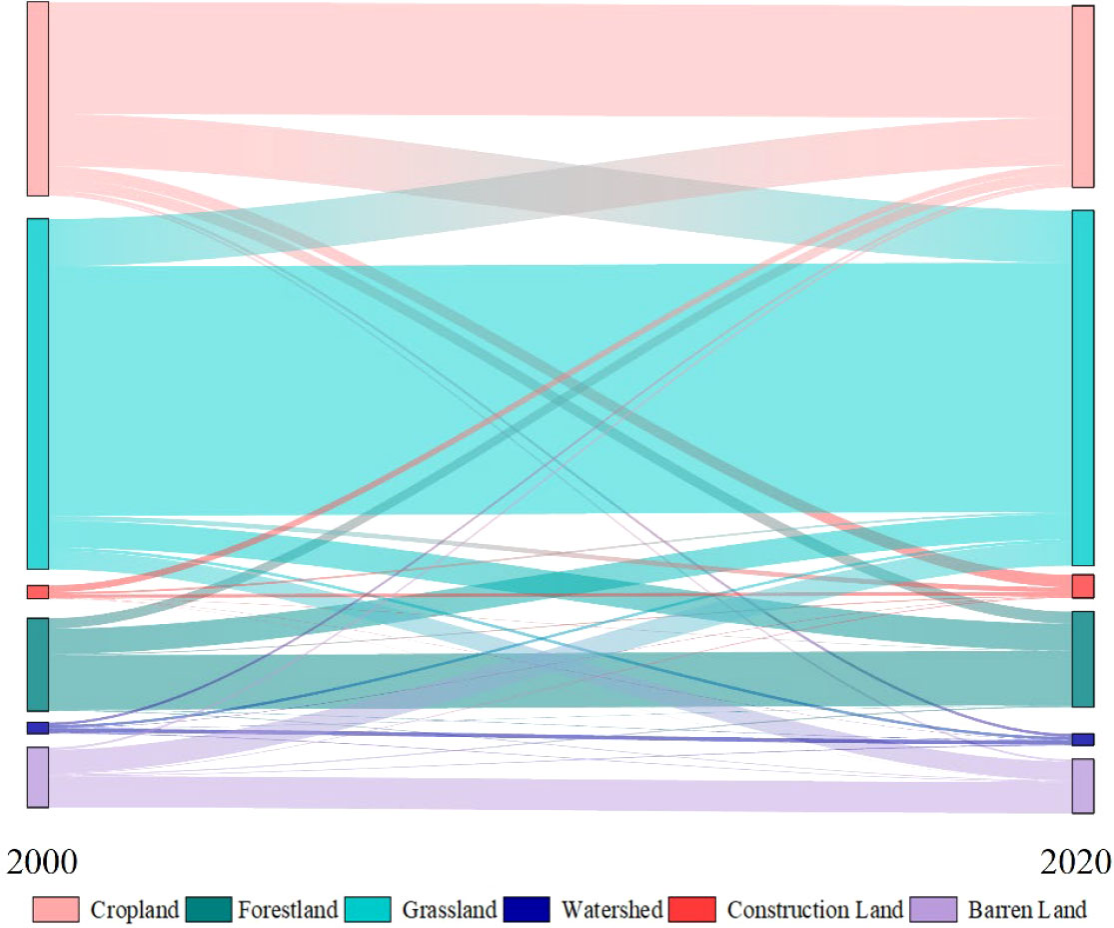
Land use transfer over the YRB from 2000 to 2020.

Rapid urbanization was the key driver of vegetation degradation (Li et al., 2018). In the LB_YRB, the multi-year average NL was the highest, whereas the NDVI trend was the lowest. Balancing urbanization and ecological protection under rapid economic development remains a critical challenge (Yuan et al., 2021). From 2000 to 2020, the total area of construction land increased from 1.71 × 10^4^ to 2.82 × 10^4^ km^2^ with a rate of approximately 65%. Approximately 1.36 × 10^4^ km^2^ cropland was transformed to construction land (**Figure 18**), where the NDVI varied by a greening rate of 0.05 per decade (**Figure 6**). Besides that, overgrazing and cultivated land expansion drove the grassland degeneration (Zhou et al., 2017). Our study stated that a total area of approximately 5.22 × 10^4^ km^2^ grassland was reduced and transformed to cropland (**Figure 18**). The average NDVI decreased from 0.68 in 2001 to 0.48 in 2020 over the corresponding region. In contrast to the source regions, the driving mechanism in the middle and lower reaches shifted to anthropogenic control. Large-scale ecological restoration projects (e.g., the Grain for Green Program) have altered the land surface boundary conditions, allowing vegetation to recover even in water-limited areas through artificial management (Feng et al., 2016). This anthropogenic forcing effectively overrides natural climatic signals, leading to a decoupling of vegetation dynamics from meteorological fluctuations in these heavily managed sub-basins (Chen et al., 2019).

#### 4.5.4 Limitations and potential studies

**Figure 19:**
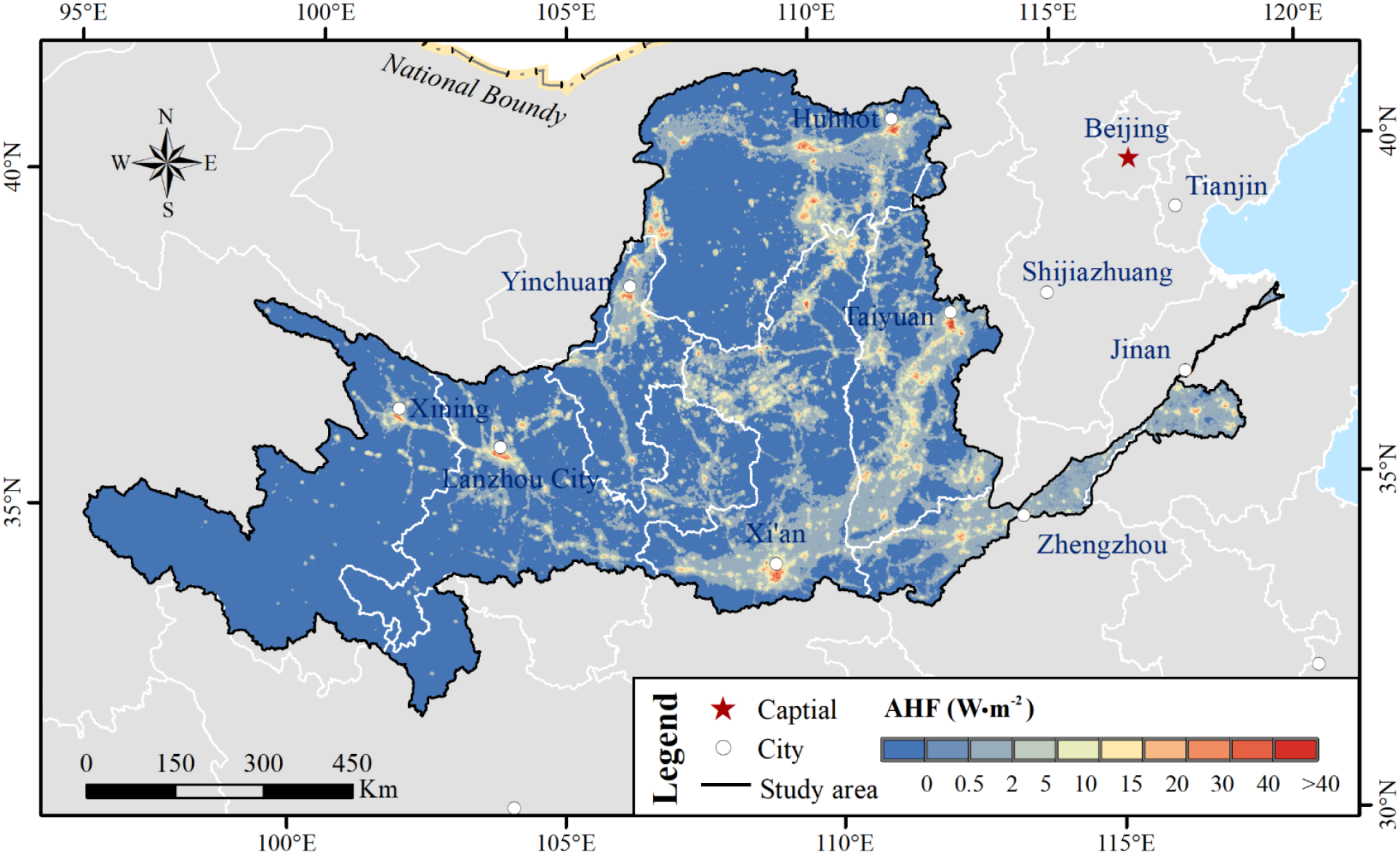
Average anthropogenic heat flux in the YRB from 2000 to 2016.

Considering the comprehensive effects of complex driving factors, the anthropogenic heat flux (AHF) of land surface (Wang et al., 2020) also influences vegetation dynamics. The spatial variations in AHF were negatively correlated with the NDVI distribution (Cao et al., 2021). Based on the AHF data with spatial resolution of 500 m × 500 m in 2000, 2004, 2008, 2012, and 2016, the average anthropogenic heat flux was 0.86 W·m^-2^ (**Figure 19**).

More than 65% of the regions in the YRB with an AHF lower than 0 were identified, and these areas generally exhibited high vegetation cover (NDVI > 0.5), accounting for 40% of such regions. Complex interactions exist between anthropogenic factors and climatic changes (Findell et al., 2017; Yin et al., 2020). Future research should quantify influences of the AHF on vegetation NDVI variations. Elucidating these anthropogenic impacts is essential to guide sustainable urban planning—for instance, quantifying urbanization-induced warming effects (e.g., surface temperature rise) on vegetation would facilitate the optimization of land use configurations to mitigate thermal stress in rapidly developing sub-basins (Ru et al., 2022). Meanwhile, natural factors also exert interactive effects on vegetation NDVI (Peng et al., 2019). Therefore, natural factors such as elevation and soil type should be evaluated for their impacts on vegetation growth. Addressing these static factors is crucial to implement “site-specific” ecological management. Clarifying the trade-off between vegetation restoration and water consumption across different landscapes will help policymakers determine the optimal vegetation carrying capacity, preventing soil drying in water-limited regions (Feng et al., 2016).

## 5 Concluding remarks

We analyzed the vegetation NDVI variations and quantified the major causal factors in the YRB from 2001 to 2020. More than 90% of the total area in the YRB exhibited a vegetation greening trend, with a mean rate of 0.056 per decade from 2001 to 2020. Regions showing statistically significant increasing trends (*p* < 0.05) accounted for approximately 50% of the YRB and were primarily located in the MB_YRB. In addition, elevation and longitude thresholds that shifted the NDVI characteristics were clarified, and NDVI decreased significantly as the latitude increased.

In the areas where NDVI showed a significant tendency (*p* < 0.05), the average contributions of the EFPR, ACTE, and HUAV were 0.012, 0.007, and 0.036 per decade, of which the homologous contribution rates were approximately 27%, 16%, and 65%, respectively. Warm and humid conditions strongly promoted vegetation growth across the YRB. ACTE was detected to affect the vegetation variations in the source regions of the Yellow River. Vegetation dynamics were mainly controlled by EFPR in the Loess Plateau and Lvliang Mountains of the MB_YRB. Anthropogenic activities, such as human land use managements, seem to be the dominant drivers in accelerating vegetation greening in the MB_YRB and LB_YRB, especially in Loess Plateau. Vegetation browning only mainly occurred in Yiluohe River Basin and LB_YRB, which are devoted to food production and urbanization. Vegetation dynamics exhibited a significant spatio-temporal heterogeneity. Overall, both climatic and anthropogenic factors were critical drivers in shaping the vegetation distribution in the YRB.

## Data Availability

The original contributions presented in the study are included in the article/supplementary material. Further inquiries can be directed to the corresponding author.
